# Impact of meteorological conditions, canopy shading and leaf removal on yield, must quality, and norisoprenoid compounds content in Franciacorta sparkling wine

**DOI:** 10.3389/fpls.2023.1125560

**Published:** 2023-05-17

**Authors:** Isabella Ghiglieno, Silvia Carlin, Gabriele Cola, Urska Vrhovsek, Leonardo Valenti, Mar Garcia-Aloy, Fulvio Mattivi

**Affiliations:** ^1^Department of Agricultural and Environmental Sciences - Production, Landscape, Agroenergy, University of Milan, Milan, Italy; ^2^Metabolomic Unit, Food Quality and Nutrition Department, Research and Innovation Center, Edmund Mach Foundation, S. Michele all’Adige, Italy

**Keywords:** vine (*Vitis vinifera* L.), leaf removal, grapevine shading, must quality, yield, sparkling wine, aroma compounds, norisoprenoid

## Abstract

Climate change is a major concern in agriculture; in grapevine production, climate change can affect yield and wine quality as they depend on the complex interactions between weather, plant material, and viticultural techniques. Wine characteristics are strongly influenced by microclimate of the canopy affecting primary and secondary metabolites of the grapevine. Air temperature and water availability can influence sugar and acid concentration in grapes and relative wines, and their content of volatile compounds such as norisoprenoids. This becomes relevant in sparkling wine production where grapes are generally harvested at a relatively low pH, high acidity, and low sugar content and where the norisoprenoids significantly contributes to the final aroma of the wine. The effect of climate change on grapevine and wine, therefore, calls for the implementation of on-field adaptation strategies. Among them canopy management through leaf removal and shading have been largely investigated in the wine growing sector. The present study, conducted over 4 years (2010-2013) aims at investigating how leaf removal and artificial shading strategies affect grape maturation, must quality and the production of norisoprenoids, analyzed using an untargeted approach, in sparkling wine. Specifically, this paper investigates the effect of meteorological conditions (i.e., water availability and temperatures) and the effect of leaf removal and shading on *Vitis vinifera* L. cv. Chardonnay and Pinot noir, which are suitable to produce sparkling wine in the DOCG Franciacorta wine growing area (Lombardy, Italy). The effect of leaf removal and shading practices on norisoprenoids has been the focus of the study. No defoliation and artificial shading treatments play an important role in the preservation of the acidity in warm seasons and this suggests calibrating defoliation activities in relation to the meteorological trend without standardized procedures. This is particularly relevant in the case of sparkling wine, where the acidity is essential to determine wine quality. The enhanced norisoprenoid aromas obtained with a total defoliation represent a further element to direct defoliation and shading strategies. The obtained results increase knowledge about the effect of different defoliation and artificial shading applications in relation to meteorological condition supporting the management decision-making in the Franciacorta wine growing area.

## 1 Introduction

Climate change means any changes in weather patterns caused by natural events and human activities during a certain period (IPCC, 2007). The temperature increase that has characterized Europe and Italy since the end of the 1980s has relevant consequences on the quantity and quality of crops, representing a major concern in agriculture (Mariani et al., 2012; Suter et al., 2021). In grape-growing for wine production, climate change can significantly affect yield and, indirectly, wine quality (Fraga et al., 2012), as they depend on the complex interactions between weather variables, plant material, and viticultural techniques (Nesbitt et al., 2016; van Leeuwen et al., 2019; Mirás-Avalos and Araujo, 2021). Air temperature, water availability, and sunlight are the most relevant factors that, influencing grape and must quality, indirectly affect wine quality. Grapes and musts characteristics are, indeed, strongly influenced by climatic conditions as changes in the microclimate of the canopy have an impact on the primary and secondary metabolites of the grapevine.

Water availability and water stress lead to different effects depending on the grapevine developmental stage, cultivar and wine target (Deloire et al., 2004; Santos et al., 2020; Mirás-Avalos and Araujo, 2021). During post-veraison excessive humidity tends to promote sugar dilution (Reynolds and Naylor, 1994), delaying the harvesting time (Tonietto, 1999) and avoiding an excessive accumulation of total soluble solids (Intrigliolo et al., 2016). In general, the water status correlates positively with berry size, total acidity, and malic acid concentration (Mirás-Avalos and Intrigliolo, 2017).

Many studies focused on the effect of temperature increases on grapevine and wine quality (Biasi et al., 2019; Santos et al., 2020). The mean air temperatures during the growing season are directly related to the length of the growing season for each variety (Jones et al., 2005); the different stages of development generally take place earlier and the time between veraison and ripening is shorter (Schultz, 2000; Jones et al., 2005). Excessively hot weather during the veraison–maturity period can significantly influence sugar accumulation (Greer, 2013), reducing the level of acidity in grapevines and, consequently, in wines (Pons et al., 2017). This becomes relevant in grapevines suitable to produce sparkling wines. In sparkling wines production grapevines are in fact generally harvested at a relatively low pH, higher titratable acidity, and lower soluble sugar content than those for table wine production (Alfonzo et al., 2020). The synthesis of these compounds tends to increase during the herbaceous phase of the berry, while their degradation is enhanced after veraison and during the final stages of ripening. Carotenoids are the precursors of the norisoprenoids, volatile compounds. Norisoprenoids can be generated by the direct degradation of carotenoids such as β-carotene and neoxanthin, and can be stored as glycoconjugates, which can then release their volatile aglycones during wine fermentation or ageing; hydrolysis is often slow and depends on various factors, such as storage duration, temperature, and pH (Winterhalter and Schreier, 1994; Deluc et al., 2009; Song et al., 2012). The olfactory perception thresholds of these compounds are very low and, therefore, they have a high sensorial impact on the wine aroma (Mendes-Pinto, 2009). The most important C13 norisoprenoids are: TDN, vitispirane, actinidols, β-damascenone, β-ionone, and E-1-(2,3,6-trimethylphenyl) buta-1,3diene (TBP-1).

Although the concentration of norisoprenoids in grapes depends on many factors, such as variety and stage of ripeness, the environmental conditions, have a fundamental role in influencing their synthesis. This is confirmed by the behavior of norisoprenoid β-damascenone that decreases in white wines in conditions of exposure to light and higher temperature (Marais et al., 1992; Kwasniewski et al., 2010).

The effect of climate change on grapes, musts and, consequently, on wine quality, therefore, calls for the implementation of on-field adaptation strategies. Among these, the management of the canopy through leaf removal and shading have been largely investigated in the wine growing sector (Downey et al., 2006; Caravia et al., 2016; Ghiglieno et al., 2020; Martínez-Lüscher et al., 2020). The influence of these agronomic practices on canopy microclimate has been widely studied (Crippen and Morrison, 1986; Jackson and Lombard, 1993; Downey et al., 2006; Ghiglieno et al., 2020). The direct solar radiation on grape caused by leaf removal and the consequent increase in berry temperature during maturation, influence berry ripening and metabolism, promoting sugar contents accumulation (Riou et al., 1994), reducing titratable acidity, and increasing malic acid degradation (Lakso and Kliewer, 1975; Conde et al., 2006; de Oliveira et al., 2019). Moreover, excessive sunlight inhibits the development of flavor and aroma components (Jones et al., 2005). Much has been written about how canopy management practices affect the content in norisoprenoids, especially TDN and vitispirane (Marais et al., 1992; Chen et al., 2017; Asproudi et al., 2018; Wang et al., 2018). Some authors (Kwasniewski et al., 2010) have indeed demonstrated that the timing of leaf removal can alter the carotenoid profile, as well as TDN and vitispirane precursors in mature Riesling grapes: if the leaves were removed one month after the berry set, they observed elevated total amounts in both must and wine. With regard to grapevine shading, although previous studies reported that artificial shading protects grapes from sunlight exposure but leads to an increase of air temperature in the fruit zone (Chorti et al, 2010), other researches carried out in Franciacorta wine growing area demonstrated that this agronomical practice also allows to decrease inner berry temperature (Ghiglieno et al., 2020). This effect of artificial on canopy microclimate makes this practice a good adaptation strategy to climate change. Many authors (Reynolds et al., 1986; Smart et al., 1990; Dokoozlian and Kliewer, 1996; Filippetti et al., 2015; Martin et al., 2016) have in fact demonstrated the effect of shading on delaying ripening and preserving acidity, in terms of both titratable acidity and malate concentration. The response of pH and potassium to artificial shading is less clear: some authors demonstrated a positive effect of shading on pH and potassium (Smart et al., 1985; Scafidi et al., 2013; Toda and Balda, 2014), while more recent studies reported that this treatment does not significantly affect these parameters (Filippetti et al., 2015).

The present 4-year (from 2010 to 2013) study aims to investigate how leaf removal and artificial shading strategies affect grape maturation, must quality and the production of norisoprenoids in sparkling wine. Wine volatiles compounds were analyzed using an untargeted approach aimed at a holistic view rather than restricted only to a list of target compounds (Cozzolino, 2016). Specifically, the paper investigates the effect of meteorological conditions (i.e., water availability and temperatures) on grapevine production and must quality. Moreover, the practice of application of different canopy shading levels *on Vitis vinifera* L. cv. Chardonnay and Pinot noir is studied to understand their effect on grapevine production and must quality as well as the on the aroma of sparkling wine in the DOCG Franciacorta wine growing area (Lombardy, Italy). Understanding the response of musts and wine quality to leaf removal and shading, in relation to the meteorological variability of each year, is useful to direct agronomical management in tackling the issue of climate change (e.g., early ripening, acidity degradation, wine aroma profile variation) that is affecting sparkling wine quality in particular. The leaf removal and shading practices on norisoprenoids compounds shall also be examined. The concentrations of these aromatic compounds tend to increase during wine aging, especially in sparkling wines, and can reach the level above the threshold of the characteristic aroma of aged wine which is sometimes detrimental for the wine quality. Since there aren’t, to our knowledge, studies that had been conducted on the modulation of norisoprenoids in relation to canopy management in finished sparkling wine, this work could be useful to understand the effect of these treatments in the final product.

## 2 Materials and methods

### 2.1 Experimental setting

This study was carried out in the winegrowing area of Franciacorta DOCG, a famous Italian sparkling wine production area located in the Lombardy Region. The experiment was repeated for four consecutive years (from 2010 to 2013) in a vineyard (45.572850° Lat, 9.967622° Lon) belonging to Azienda Agricola Castello Bonomi Tenute in Franciacorta. This company is located in the southern part of the Franciacorta region (**Supplementary Figure 1**).

The soil of experimental vineyard is grass-covered and characterized by a loamy texture, high total organic carbon content and CEC (Cation Exchange Capacity). The vineyard, with row orientation from north to south, was planted in 2004 with two international *Vitis vinifera* L. cultivars, Chardonnay (clone ENTAV-INRA^®^ 96) and Pinot noir (clone 292), both grafted onto Kober 5BB rootstock. Both cultivars are cordon trained.

The experimental plan included five different treatments: a control test without defoliation and shading (ND), a test where about six basal leaves, equal to about 35% of total leaf area, were removed, east and west side (TD), and three different systems adopting shading nets applied along the bunch zone; two of the shaded treatments were defoliated as for TD and covered with one layer of shading net (TD1) or two layers of shading net (TD2), while a third treatment was covered by only one layer of shading net, but not defoliated (ND1L).

The treatments were replicated on both the Chardonnay and Pinot noir cultivars. All treatments were set in two replicates represented by two rows of the vineyard for each cultivar. Each treatment was extended for 25 vines for each of the two replicates. All treatments took place at about 20% veraison, corresponding to July 19th, July 14th, July 19th, July 29th, respectively, in 2010, 2011, 2012 and 2013. Both manual leaf removal and shading net application were carried out along the bunch zone; shading was realized through a UVstabilized polyethylene net of approximately 95 g m^-2^ (shading net OF50N supplied by Retes srl). The transmittance of global solar radiation of the single layer and double layer nets was preliminarily tested in order to evaluate the percentage of global solar radiation passing through the nets. A reduction by 50% and 70% was detected for single- and double-layer shading nets, respectively.

### 2.2 Meteorological data and agrometeorological indices

During the four years, weather conditions were monitored by means of the weather station of Rovato (BS) of the Agrometeorological Network of the Province of Brescia, which is located at a distance of 5 km from the experimental vineyard.

To provide a more precise description of the thermal conditions in the experimental vineyard a four-channel data logger (Onset HOBO U12) equipped with an air temperature sensor (thermistor Onset HOBO S-THC), protected by a solar shield was placed in the vineyard.

Precipitation data recorded by the Rovato weather station and temperature data from the vineyard thermometer were used to characterize the general meteorological features of the four years of investigation and to calculate thermal indices, the grapevine water balance and the derived water stress indices, as described further below.

#### 2.2.1 Water stress

In order to provide a description of the relationship between plant growth and water resources, a single-layer reservoir model with a daily time step was created (Cola et al., 2014), considering a reference soil reservoir of 120 mm. The daily reference evapotranspiration (ET0) was calculated by means of the Hargreaves-Samani method and the daily maximum evapotranspiration of grapevine (ETM) was obtained by means of a dynamic crop multiplicative coefficient (Kc), as a function of the phenological stage of the plant, modelled on the base of air temperature-based course (Mariani et al., 2013). The daily real grapevine evapotranspiration (ETR) was obtained by means of the water limiting factor (WLFR) response curve, that relates the soil water content with the plant stress. When the soil water level is between the field capacity and the easily available water limit, the plant does not face stress, WLFR is equal to 1 and ETR=ETM. Otherwise, when the water content moves above the field capacity up to saturation or when it moves from the easily available water limit down to the wilting point, WLFR linearly decreases toward 0. As a consequence, the stress increases and ETR decreases.

In this work, the seasonal water stress (WSTR) was obtained cumulating the daily values of 1-WLFR, so that a day without water stress weights 0 and a day with maximum water stress weights 1. Additionally, the cumulated evapotranspiration deficit (ΔET) was obtained, on the base of the daily difference [mm] between ETM and ETR throughout the season.

#### 2.2.2 Thermal regime

In order to describe the air thermal condition during fruit development and ripening, thermal resources and limitations were obtained on the basis of the air temperature, applying the Normal Heat Hours (NHH) response curve (Mariani et al., 2012; Mariani et al., 2013; Cola et al., 2020). This method tries to overcome the overestimation of high temperature in terms of plant development that characterizes the classic growing degree-days approach of the Winkler Index (Amerine and Winkler, 1944) in which a very warm day is translated into a high value of the index, meaning a strong positive contribution to plant growth when high air temperature values are detrimental for the plant development.

Indices were calculated considering meteorological conditions along the berry development period (i.e., from berry set stage to harvest) and specifically: from June 1^st^ to August 26^th^ for 2010; from May 20^th^ to August 1^st^ 2011; from May 29^th^ to August 8^th^ 2012; from June 6^th^ to August 22^nd^ 2013.

NHH indicates the total hourly thermal resources useful for berry maturation from fruit set to physiological maturity (Cola et al., 2017; Cola et al., 2020) and represent thermal resources. Low Heat Hours (LHH) represents the stress caused by hourly temperatures below the optimal level and High Heat Hours (HHH) is the stress caused by hourly temperatures above the optimal level.

More in detail, air temperature is weighed on the base of four parameters, namely, low cardinal - LC, low optimal cardinal – LOC, upper optimal cardinal – UOC and upper cardinal UC, respectively:

* When the hourly air temperature is below LC: LHH=1, NHH=0, HHH=0, meaning that the whole hour was spent by the plant under stressing conditions caused by low temperatures.
* When the hourly air temperature is above UC: LHH=0, NHH=0, HHH=1 meaning that the whole hour was spent by the plant under stressing conditions caused by high temperatures.
* When the hourly air temperature sits between LOC and UOC: LHH=0, NHH=1, HHH=0 meaning that the whole hour was spent by the plant under restful conditions and the development was non limited.
* Between LC and LOC the low temperature stress decreases linearly moving from LC to LOC, so that LHH moves from 1 to 0 and NHH from 0 to 1 (always with LHH+NHH=1 and HHH=0).
* Between UOC and UC the high temperature stress increases linearly moving from UOC to UC so that NHH moves from 1 to 0 and HHH from 0 to 1 (always with NHH+HHH=1 and LHH=0).

The parametrization of the four parameters (LC=12°C, LOC=24°C, UOC=26°C, UC=33°C) proved to perform very well in describing the development of several cultivars (Cabernet-Sauvignon, Chardonnay, Barbera and Georgian cultivars Mtsvane Kakhuri, Rkatsiteli, Ojaleshi and Saperavi) (Mariani et al., 2013; Cola et al., 2014; Cola et al., 2017).

In this work, NHH, LHH and HHH were cumulated from fruit-set to harvest to describe the thermal course of the season. Phenological stages of each plot were determined on the base of weekly phenological monitoring.

Additionally, the stress caused by heat waves (33STR) was obtained by counting the number of days between fruit set and physiological maturity with a maximum temperature above 33°C (Cola et al., 2020).

### 2.3 Yield components and the composition of must

With the aim to compare musts and wine composition at a standard potential alcohol degree, for both cultivars and all treatments, the harvesting time was established on the basis of the strategy adopted by the wine company for the specific year and ranging from 10 to 10.7% of potential alcohol.

The evolution of ripening for both Chardonnay and Pinot noir was monitored weekly (maintaining an interval of 7-5 days between each sampling), in order to identify the relevant harvesting time for each treatment.

Experimental harvesting was organized by selecting 5 grapevines from the two 25 vine replicates for each treatment, for a total of 10 vines per treatment (ND, TD, TD1, TD2 and ND1L). For each grapevine, the average bunch weight (ABW) was calculated counting the number of bunches and weighing the entire grapevine production. A sample of three bunches was collected from each grapevine to check must quality. These samples were then crushed and the total soluble solids concentration (TSS), pH, titratable acidity (TA) and malic acid (MA) concentration were analyzed in the resulting musts. These measurements were determined using a traditional handheld refractometer for soluble solids concentration, a Crison compact titrator analyser for both pH and TA, and the enzymatic method (Hyperlab wine analyser) to determine MA concentration.

### 2.4 Wine production

About 100 kg of grapes were harvested for each cultivar and treatment upon reaching the standard potential alcohol. Grapes were harvested in small boxes weighing maximum 15 kg, and stocked in a cold room at about 10°C. Microvinifications was then initiated according to a standardized protocol. The wine-making protocol was organized in different steps described in **Supplementary Figure 2**.

The wine so obtained was stocked in 15 l demijohns and periodically monitored for free sulfur content, using the WineScan™ SO2 (FOSS) instrument, to avoid undesired malic fermentation. After about five months the wine was stabilized by adding 10 ml hl^-1^ silica sol and 1 ml hl^-1^ gelatin. The wine was then bottled using 0.75 l bottles (for a total of 18 bottles); nine bottles were then stocked and 0.3 g l^-1^ of yeast and 22 g l^-1^ of sugar were added to the other nine bottles to induce refermentation, thus allowing to produce a final sparkling wine. After this last step, all bottles were stocked and manually disgorged, after a manual remuage to remove the residue of yeasts without any further addition. Disgorgement was performed for all wine samples at the time of the volatile aroma compounds analysis in 2016 and 3 different bottles for each treatment were used as replicates.

### 2.5 Analysis of volatile aroma compounds by GC×GC-TOF-MS

All the analyses were carried out following the method described in our previous works (Carlin et al., 2016; Carlin et al., 2022). In order to monitor instrument stability, pooled quality controls (QC) consisting of equal proportion of each sample were placed at the beginning of the run (n=5) and thereafter every 10th sample as a common practice in metabolomics studies (Arapitsas et al., 2016). For each sample, 2 ml of wine with 50 μl of internal standard solution (2-octanol in ethanol at a concentration of 1 mg l^-1^) was mixed with 1 g NaCl in a 20 ml headspace vial. A Gerstel MultiPurpose Sampler autosampler (Gerstel GmbH & Co. KG Mülheim an der Ruhr Germany) with an agitator and SPME fiber 2 cm (50/30 DVB/CAR/PDMS), from Supelco Merk KGaA (Darmstadt, Germany), was used to extract the volatiles from the sample vial headspace. The sample was pre-incubated for 5 min at 35°C. Adsorption lasted 20 min, at the same temperature, Then, desorption took place in the injector in splitless mode (4 min) at 250°C. The fiber was then reconditioned for 10 min at 250°C. The GC×GC system was the Agilent 7890 A (Agilent Technologies, Santa Clara, CA). Injections were performed in splitless mode. Equipped columns were the VF-Wax column (100% polyethylene glycol; 30 m × 0.25 mm × 0.25 μm, Agilent J&W Scientific Inc., Folsom, CA) as the 1^st^ dimension and Rxi-17Sil MS 1.50 m × 0.15 mm × 0.15 μm, Restek Bellefonte, USA) as the 2^nd^ dimension. The GC system was equipped with a secondary column oven and non-moving quadjet dual-stage thermal modulator. The injector/transfer line was maintained at 250°C. Oven temperature program conditions were as follows: initial temperature of 40°C for 4 min, programmed at 6°C min^−1^ at 250°C, where it remained for 5 min. The secondary oven was kept at 5°C above the primary oven throughout the chromatographic run. The modulator was offset by +15°C in relation to the secondary oven; the modulation time was 7 s and 1.4 s of hot pulse duration. Helium (99.9995% purity) was used as carrier gas at a constant flow of 1.2 ml min^-1^. The MS signal was obtained with a Pegasus IV time-of flight mass spectrometer (Leco Corporation, St. Joseph, MI) with electron ionization at 70 eV and the ion source temperature at 230°C, detector voltage of 1317 V, mass range of m/z 35–450 and acquisition rate of 200 spectra s^−1^.

For GC×GC-MS data, LECO ChromaTOF Version 4.22 software was used for all acquisition control, data processing and Fisher ratio calculations. Automated peak detection and spectral deconvolution with a baseline offset of 0.8 and signal-to-noise of 100 were used during data treatment. Before proceeding with the data analysis a quality control of the data sets, checking the distribution of the QC injections was carried out (Arapitsas et al., 2016). The identification of the wine volatile compounds was achieved by comparing the mass spectrometric information for each chromatographic peak with NIST 2.0, Wiley 8 and the FFNSC 2 mass spectral library (Chromaleont, Messina, Italy), with a library similarity match factor of 750 and comparing also the experimental linear temperature retention index (LTPRI) with retention indices reported in the literature for 1D-GC (VCF Volatile Compounds in Food 16.1 database). Retention data for a series of n-alkanes (C10–C30), under the same experimental conditions employed for chromatographic analysis of wine volatiles, were used for experimental LTPRI calculation. The content of norisoprenoids were expressed as peak areas.

### 2.6 Statistical analysis

In the statistical analysis, Chardonnay and Pinot noir were kept separated in relation to the different composition of their grapes and their different sensory characterization in wines (Herrero et al., 2016).

The effect of the year was initially investigated for variables related to ABW and musts quality through an analysis of variance (ANOVA) and Siegel-Tukey’s *post-hoc* test using the SPSS software (Statistical Package for Social Science) (IBM Corp, 2021).

The relationships between year and agrometeorological indices and AWB and must quality were also investigated through a principal component analysis (PCA) on autoscaled data (i.e., mean-centered and divided by the standard deviation of each variable), through R software (R Core Team, 2022). In the PCA analysis for each cultivar, five points for each year, corresponding to the average value of the single treatment in a specific year, were considered. This approach was used because one single value of agrometeorological indices was available for each year without having the possibility to differentiate between treatments.

Additional ANOVA and Siegel-Tukey’s *post-hoc* test were performed to determine the influence of the treatment on variables related to ABW and musts quality (TSS, TA, MA, pH). In this analysis, both the cultivars and the four years were considered separately. The decision to process the different years separately was determined by the meteorological differences in the individual years and the effect on the AWB and must quality revelead by the results obtained from the analysis of the relationship between year and these variables.

Considering the limited number of biological replicates used for the untargeted volatile compounds analyses (i.e., 3 replicates for each cultivar, treatment, and year) a different approach was outlined. In this case, it was checked whether there were any statistically significant differences associated with each treatment compared to the untreated thesis (ND) by means of a linear model on log-scaled data using R software (R Core Team, 2022).

## 3 Results

### 3.1 Meteorological characterization

**Table 1** shows the monthly variation of temperature and precipitation by season, respectively, while the agrometeorological indices are shown in **Table 2**.

**Table 1:** Monthly average and standard deviation of maximum and minimum temperature and monthly total precipitation for the seasons 2010-2013.

Month	Air Temperature [°C]								Monthly Precipitation [mm]			
	Minimum				Maximum							
	2010	2011	2012	2013	2010	2011	2012	2013	2010	2011	2012	2013
Jan	-1.3 (2.2)	-0.6 (2.2)	-1.4 (2.7)	0.4 (1.7)	4.9 (2.8)	6.1 (2.3)	8.8 (3.8)	7.3 (3.8)	32	29	26	59
Feb	0.9 (3.1)	1.7 (2.7)	-2.8 (4.8)	-0.1 (2.0)	8.9 (3.2)	11.4 (3.0)	7.3 (6.8)	7.6 (3.1)	93	62	12	61
Mar	3.7 (4.4)	5.2 (3.5)	6.8 (2.6)	3.8 (2.3)	13.5 (4.8)	14.7 (4.2)	19.3 (3.9)	11.3 (3.6)	49	46	11	137
Apr	8.1 (3.2)	10.1 (2.0)	7.9 (2.6)	9.4 (3.3)	19.9 (4.2)	23.0 (3.3)	17.8 (3.7)	18.5 (5.0)	71	13	146	96
May	12.2 (2.1)	12.9 (3.1)	12.1 (3.0)	10.9 (2.3)	23.1 (3.9)	26.5 (3.2)	24.5 (4.0)	22.1 (3.3)	165	44	128	180
Jun	16.8 (2.9)	16.0 (1.5)	17.4 (2.8)	15.5 (3.2)	28.7 (3.7)	26.9 (2.9)	30.0 (3.8)	28.7 (3.9)	94	122	33	53
Jul	19.2 (2.8)	16.7 (2.5)	18.6 (1.9)	19.2 (1.9)	32.0 (2.6)	28.7 (2.2)	32.2 (1.9)	32.1 (1.9)	91	79	39	50
Aug	16.7 (2.8)	18.2 (2.3)	19.7 (2.4)	17.5 (2.3)	29.2 (3.2)	31.0 (2.3)	33.5 (3.2)	30.9 (3.2)	154	93	35	109
Sep	13.1 (2.4)	16.4 (2.4)	14.4 (2.6)	14.3 (2.5)	24.8 (3.0)	28.3 (2.4)	25.9 (3.1)	26.1 (3.2)	105	143	125	23
Oct	8.1 (3.3)	8.5 (3.9)	10.5 (3.5)	11.8 (2.4)	17.5 (3.4)	20.2 (5.5)	19.9 (4.5)	19.0 (2.3)	128	60	146	91
Nov	6.0 (3.9)	4.6 (4.6)	6.8 (2.5)	5.6 (4.5)	12.6 (3.6)	13.9 (2.9)	14.3 (2.5)	13.4 (4.2)	178	63	140	111
Dec	-1.7 (3.7)	1.6 (3.0)	-0.9 (2.5)	1.4 (3.0)	5.3 (3.5)	9.4 (2.3)	7.2 (3.6)	10.9 (2.7)	151	32	68	62

**Table 2:** Environmental indices for the characterization of the four seasons.

	WSTR	ΔET	NHH	LHH	HHH	33STR
2010	3.50	4.30	1276	412	324	65
2011	2.86	8.62	1151	468	140	2
2012	24.83	79.18	1020	288	315	93
2013	18.95	52.91	1075	315	321	99

The year 2010 was characterized by optimal water conditions. The scarce precipitation of the winter period (Jan-Mar) was preceded by good precipitations in the 2009 Oct-Dec period and followed by abundant spring precipitations. From a thermal point of view, 2010 showed lower maximum temperatures during spring than 2011 and 2012, resulting in the highest NHH value in the series. However, the indices for this year were strongly affected by a longer interval between fruit set and harvest, than in the other three years. Low temperature stress (LHH) was the second highest over the four years and high temperature stress (HHH) was the highest. The 33°C limit was exceeded for 65 days during the season.

The year 2011 was still characterized by very low water stress, thanks to the refilling of the water reservoir during winter and the good distribution of precipitation over the months. As compared to other years, low temperatures caused a high level of NHH (second highest), the highest value of low temperature stress (LHH) and the lowest value of high temperature stress (HHH), confirmed by only 2 days with maximum temperatures above 33°C.

The year 2012 was characterized by the highest water stress value over the four years (as shown by WSTR and ΔET values), mainly due to the limited precipitation during June, July and August that led to a long period of a limited water soil content. From a thermal point of view, the year 2012 featured the lowest levels in terms of NHH accumulation (though very close to 2013), and LHH and the highest level in HHH (thermal excess, with 93 days of maximum temperature above 33°C).

The year 2013 was very similar to 2012, with a certain amount of water stress (second highest in both indices), the second lowest in NHH and LHH, and the second highest in HHH and the first in the number of days with a maximum temperature above 33°C (99 days).

Considering the thermal behavior in the four seasons in relative terms, in 2010 the percentages of NHH, LHH and HHH versus their total (NHH+LHH+HHH) were 63.85, 18.23 and 17.92 respectively. 2012 and 2013 showed a similar behavior with 62.85, 17.78, 19.38 and 62.81, 18.41, 18,77 respectively. The year 2011, with 65.41, 26.62, 7.97 showed a strongly different repartition, again emphasizing its unique environmental conditions.

### 3.2 Year effect

#### 3.2.1 Average bunch weight

**Figure 1:**
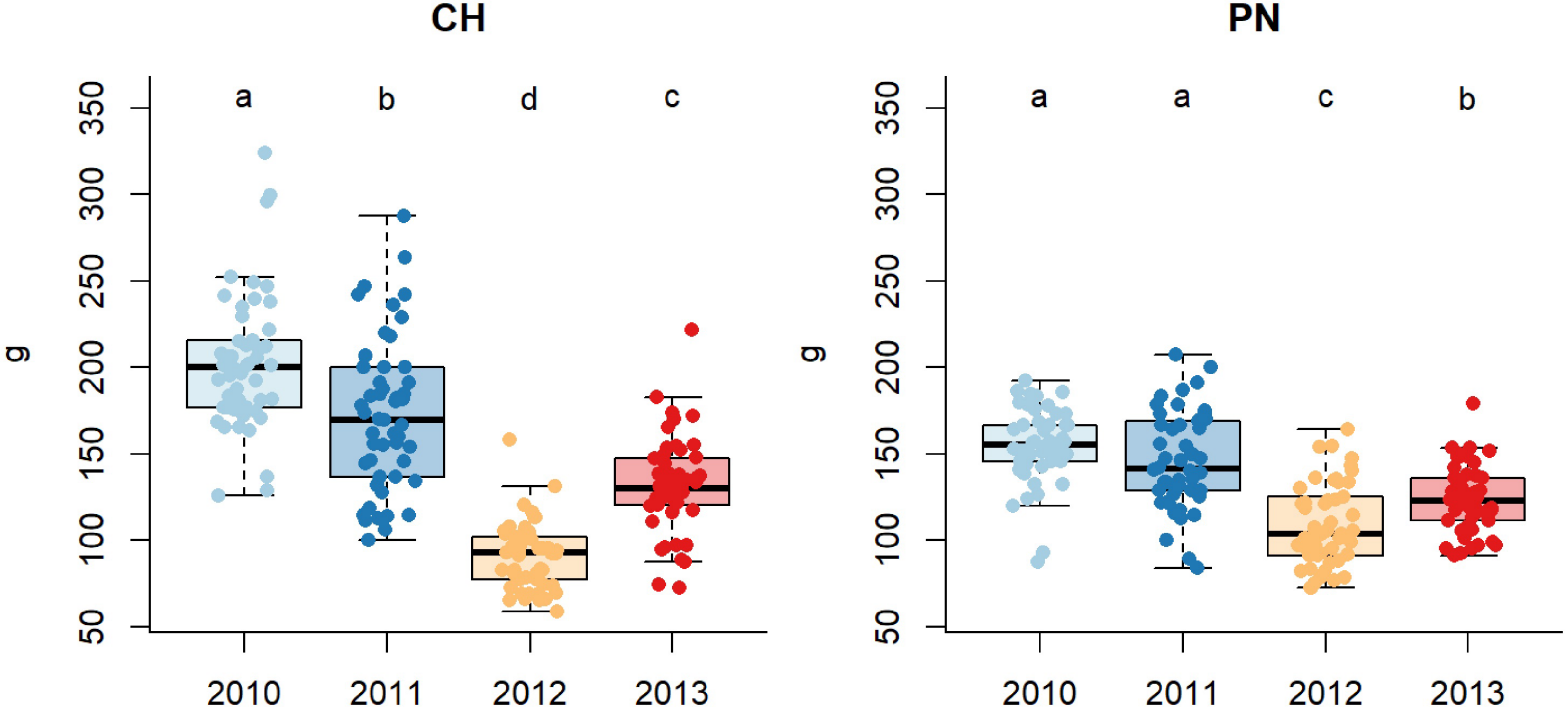
Boxplots showing average bunch weight (ABW in grams) over the 4 different vintages for Chardonnay (CH) and Pinot noir (PN): different letters indicate that values are significantly different at p < 0.05 obtained by one-way ANOVA and Siegel-Tukey’s *post-hoc* test.

The ABW varies over the years equally for both cultivars (**Figure 1**). The year 2012 showed a significantly lower value of ABW than that of the other years. The year 2013 showed an intermediate response, while 2011 and 2010 recorded higher values.

#### 3.2.2 Harvesting time

The four years were characterized by different harvesting times due to varying ripening rates. The year 2010 was characterized by a delayed maturation. Harvesting times ranged from 24/08 to 2/09 for the cv. Chardonnay, and from 26/08 to 6/09 for Pinot noir. The delay in ripening was further confirmed by the lower average sugar content reached at harvesting time (i.e., 18°Brix). The years 2011 and 2012 had the earliest vintages, while year 2013 can be considered in between, having recorded harvesting times from 28/08 to 2/09 for Chardonnay and from 20/08 to 24/08 for Pinot noir.

#### 3.2.3 Must characterization

**Figure 2:**
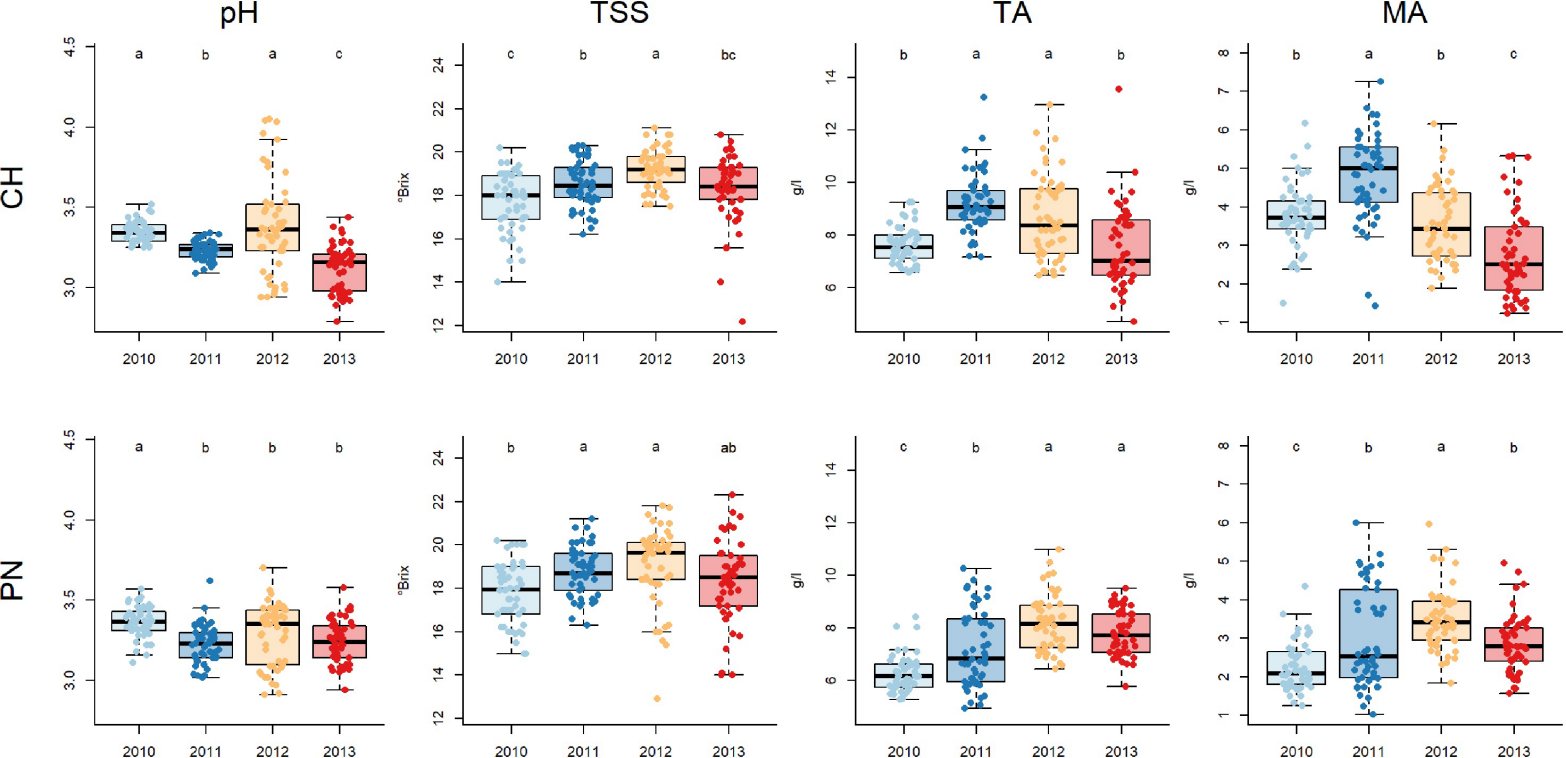
Boxplots showing data of total soluble solids concentration (TSS), titratable acidity (TA), malic acid (MA) and pH over the 4 different vintages for Chardonnay (CH) and Pinot noir (PN): different letters indicate that values are significantly different at p < 0.05 obtained by one-way ANOVA and Siegel-Tukey’s *post-hoc* test.

The results obtained in must characterization (**Figure 2**) underlined a similar response concerning TSS when comparing Chardonnay and Pinot noir, while some differences were observed in terms of acidity and pH.

TSS was higher in 2012, while 2010 recorded the lowest value; in the case of Pinot noir no significant differences were recorded between 2012 and 2011.

The year 2012 also showed a high TA value for both cultivars, as in 2011 for Chardonnay and in 2013 for Pinot noir. The year 2010 recorded the lowest TA value for Pinot noir, while no significant differences were recorded compared to the 2013 value for Chardonnay.

Results related to MA were similar to TA: for Pinot noir, the year 2012 differed significantly from 2010, recording the highest and the lowest values, respectively; in the case of Chardonnay the highest value was recorded in 2011 and the lowest value in 2013. pH values were high in 2010 for both cultivars, as in 2012 for Chardonnay. Pinot noir showed no differences over 2011, 2012 and 2013 for this variable, while Chardonnay showed the lowest value in the case of 2013.

#### 3.2.4 Years general responses

**Figure 3:**
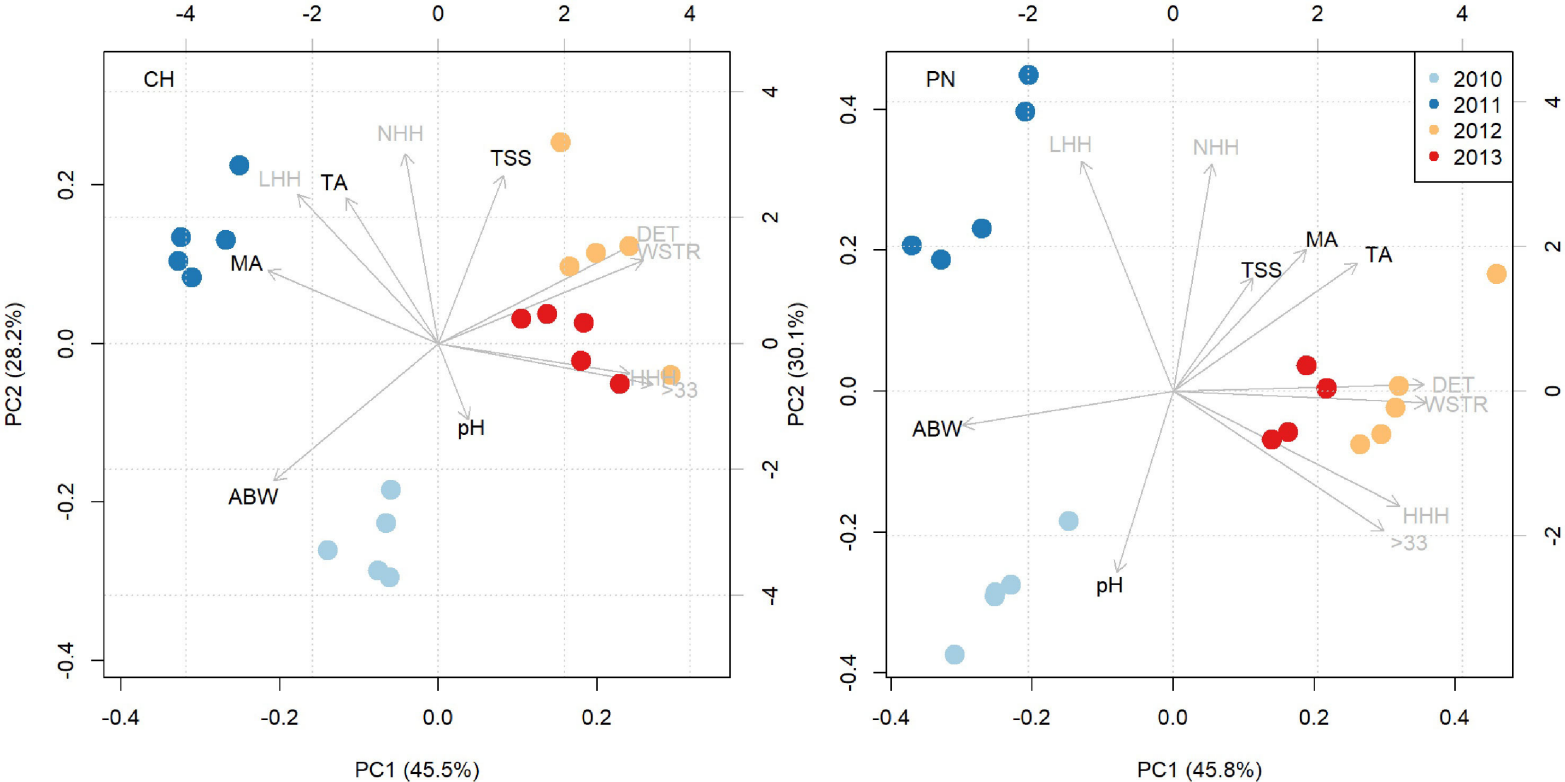
Biplots showing the distribution of data along of the two first principal component dimensions for Chardonnay (CH) and Pinot noir (PN). Stress caused by heat waves (33STR), High Heat Hours (HHH), daily evapotranspiration deficit (ΔET), seasonal water stress (WSTR), Normal Heat Hours (NHH), Low Heat Hours (LHH), average bunch weight (ABW), total soluble solids concentration (TSS), titratable acidity (TA), malic acid (MA), pH are shown as vectors. Data from a single year is identified by different colored dots. Each dot represents the average value of the single treatment in a specific year.

The biplots obtained from PCA analysis allowed to visualize the distributions of the data collected for Chardonnay and Pinot noir during the four-year 2010-2013 period (**Figure 3**). The pattern in the sample distribution highlighted a differentiation, especially between the years 2010, 2011 and 2012-2013. In both PCAs, the first two dimensions explained around 75% of variance. Vectors made it possible to display the inner relationship between years and variables (i.e., meteorological variables and ABW and must quality). Supporting the results described in the previous Section 3.1, the years 2012 and 2013 were associated, for both cultivar, to stress caused by water scarcity, higher evapotranspiration (ΔET) and temperatures (HHH and 33STR), while 2011 was associated with lower temperatures (LHH). The pattern in the distribution of years concerning vectors related to average bunch weight (ABW) and musts quality variables (pH, TSS, TA, MA) confirms the findings previously reported in Sections 3.2.1 and 3.2.3.

### 3.3 Treatment effect

#### 3.3.1 Average bunch weight

**Figure 4:**
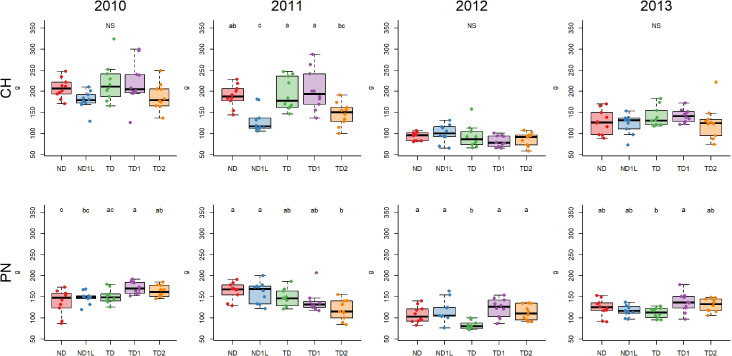
Boxplots showing average bunch weight (ABW in grams) for each treatment considering 4 years, for Chardonnay (CH) and Pinot noir (PN): different letters indicate that values are significantly different at p < 0.05; NS indicates no significant differences among treatments at p < 0.05 obtained by one-way ANOVA and Siegel-Tukey’s *post-hoc* test.

The results obtained for Chardonnay ABW by comparing treatments showed no homogeneous response over the years (**Figure 4**). In general, no significant differences were found between treatments. A significant difference emerged in 2011 between the less shaded (i.e., TD and TD1) and high shaded (i.e., ND1L, and TD2) treatments. Pinot noir recorded a generally negative effect of defoliation without shading (TD) on ABW in 2012 and 2013 (**Figure 4**). The years 2010 and 2011 showed a different response, reporting an intermediate value for TD. Other treatments related to defoliation and shading (TD1 and TD2) showed the opposite response in 2010 and 2011, resulting in a higher ABW in 2010 compared to no defoliated treatment (ND and ND1L) and a lower ABW in 2011 compared to ND and ND1L.

#### 3.3.2 Must characterization

##### 3.3.2.1 Chardonnay

**Figure 5:**
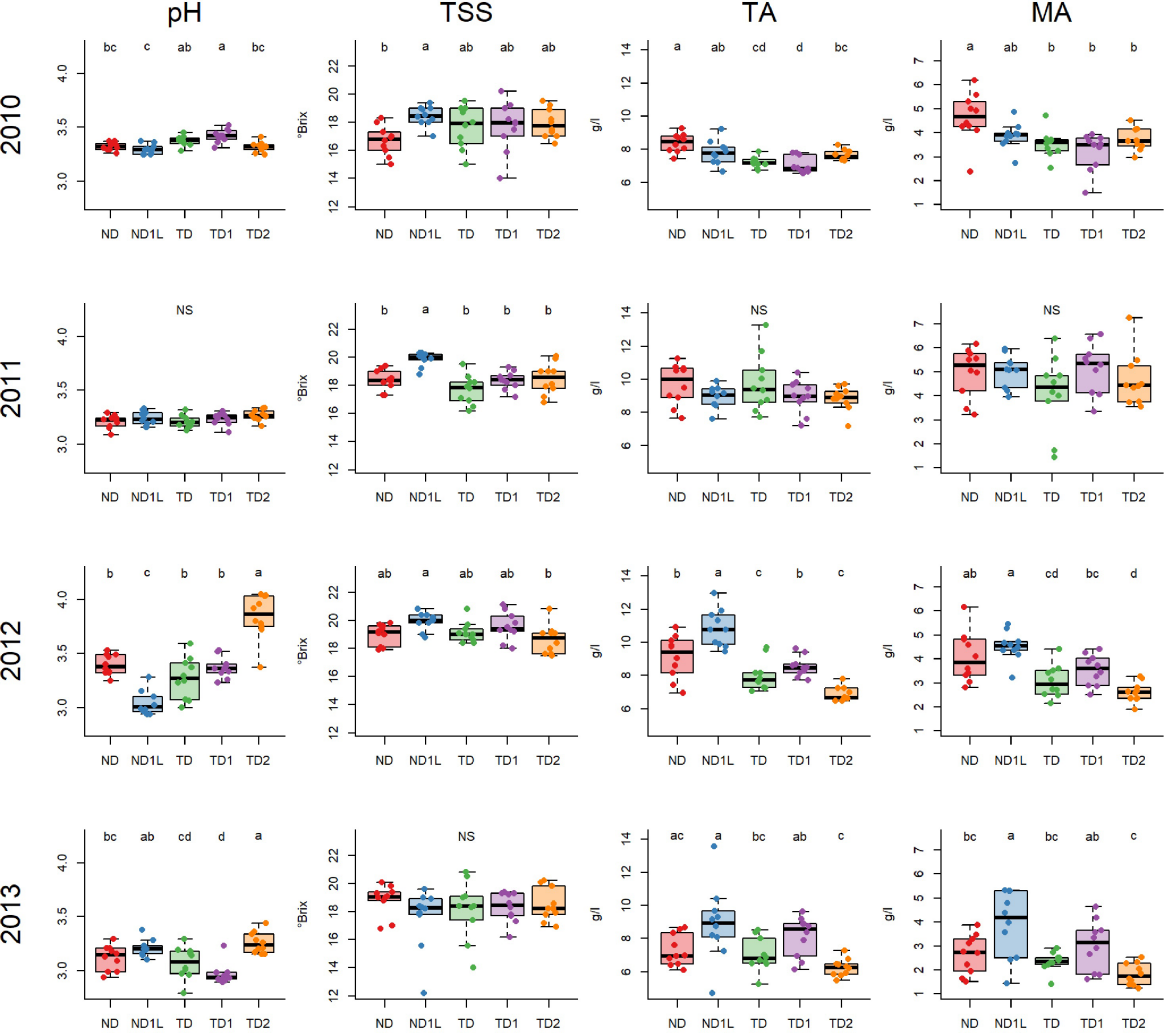
Boxplots showing total soluble solids concentration (TSS), titratable acidity (TA), malic acid (MA) and pH for each treatment considering 4 years, 2010, 2011, 2012, 2013, for Chardonnay: different letters indicate that values are significantly different at p < 0.05; NS indicates no significant differences among treatments at p < 0.05 obtained by one-way ANOVA and Siegel Tukey’s *post-hoc* test. Each column is referred to one variable (each variable is reported in the upper part of the graph); each row is referred to one year (each year is reported in the left part of the graph).

Concerning sugar concentration (TSS), no significant differences were highlighted in 2013, while differences among treatments emerged in the other years (**Figure 5**). Specifically, the ND1L generally recorded high TSS values compared to other treatments. This can be observed by comparing ND1L and ND in 2010, ND1L and all other treatments in 2011 and comparing ND1L and TD2 in 2012. Titratable acidity (TA) and malic acid showed, in general, the same response (**Figure 5**). Not defoliated treatments (ND and ND1L) tended to preserve the acidity better than defoliated treatments (TD, TD1 and TD2). This can be seen in all the years considered, except in 2011, when no significant differences were found. The efficiency of the not defoliation and shading effect on acidity preservation is more evident in 2012 and 2013; in these years, the not defoliated and shaded treatment (ND1L) recorded the highest level of both titratable acidity and malic acid concentration. Significant differences can be observed in 2010, 2012 and 2013 for pH, even though the response was generally not uniform between years (**Figure 5**). In 2012 and 2013 the highest values recorded concerned TD2, while in 2010 the highest values can be associated to TD1.

##### 3.3.2.2 Pinot noir

**Figure 6:**
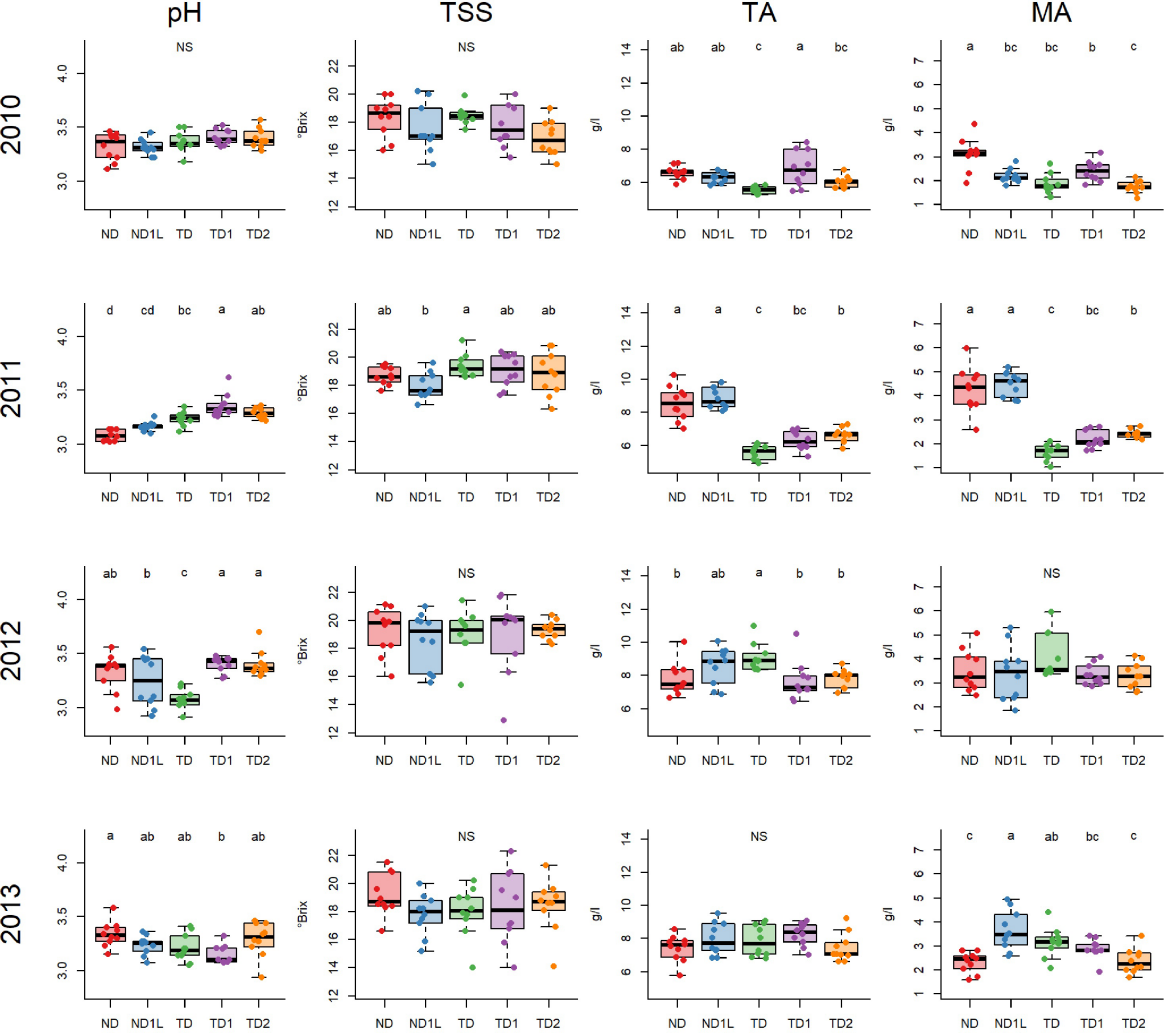
Boxplots showing mean and unit variance of total soluble solids concentration (TSS), titratable acidity (TA), malic acid (MA) and pH for each treatment considering 4 years, 2010, 2011, 2012, 2013, for Pinot noir: different letters indicate that values are significantly different at p < 0.05; NS indicates no significant differences among treatments at p < 0.05 obtained by one-way ANOVA and Siegel-Tukey’s *post-hoc* test. Each column is referred to one variable (each variable is reported in the upper part of the graph); each row is referred to one year (each year is reported in the left part of the graph).

**Figure 6** shows the results obtained for Pinot noir musts characterization. Sugar concentration (TSS) showed no significant differences among treatments with the exception of year 2011. In this year, the defoliated treatment recorded a higher sugar concentration than ND1L. Regarding titratable acidity (TA) and malic acid concentration (MA), the treatments revealed similar trends in 2010 and 2011. Considering these two years, the totally defoliated (TD) and defoliated and shaded (TD1 and TD2) treatments recorded the lowest values in both titratable acidity and malic acid concentration, with the exception of the results obtained for TD1, which in 2010 were similar to those obtained for the not defoliated treatments (ND and ND1L). Significant differences were highlighted only in the case of titratable acidity in 2012 and in the case of malic acid concentration in 2013; in these cases, ND1L and TD recorded the highest values. pH didn’t show significant differences in 2010. As described in the case of Chardonnay, the trend observed for pH was not homogeneous over the years.

#### 3.3.3 Effect on sparkling wine norisoprenoids

**Figure 7:**
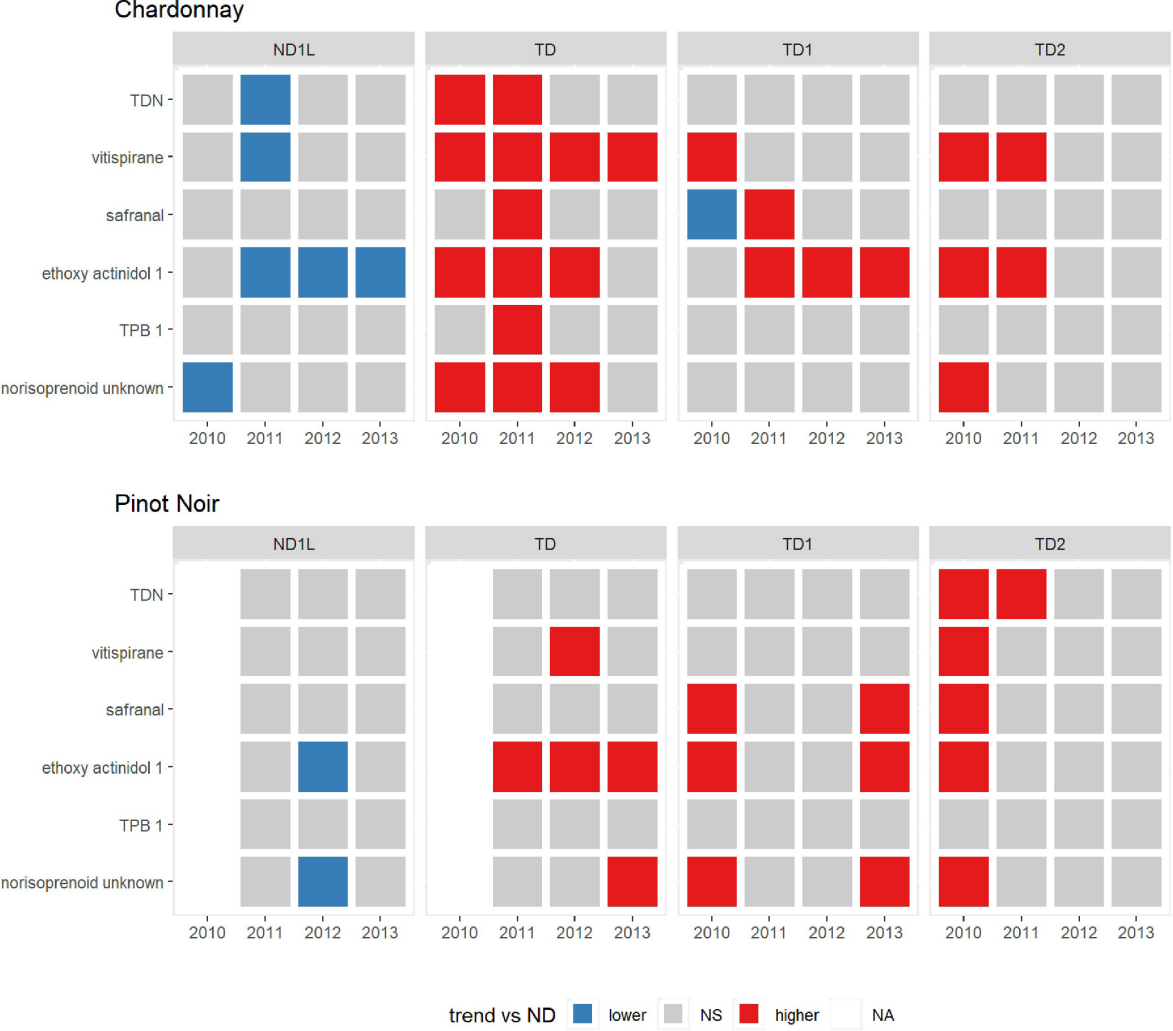
Matrix plot colored cells showing the statistically significant comparison of some norisoprenoids using a red color when mean value of treatment was statistically higher than the mean value of the reference treatment (ND) or a blue color in the opposite case over the 4 years (2010, 2011, 2012, 2013) for Chardonnay and Pinot noir. NS means not significant, NA means not available data.

As indicated in the Section 2.6 statistical analyses performed on norisoprenoids abundances were restricted to check whether, within a specific cultivar and year, there were statistically significant differences associated to each treatment compared to reference treatment, i.e. the not defoliated thesis (ND). In order to display the data, a matrix plot was performed (**Figure 7**) by indicating with colored cells the statistically significant comparisons, using a red color when the treatment mean value was statistically higher than the mean value of the reference treatment, or a blue color in the opposite case (grey cells indicate those cases of no statistical significance).

##### 3.3.3.1 Chardonnay

In the 2010 vintage, TDN, vitispirane, ethoxy actinidol 1 and the unknown norisoprenoid were present in greater quantities in the total defoliated treatment (TD), the unknown norisoprenoid was also greater in the TD2 treatment, while the lowest contents were observed in ND1L treatment. Vitispirane was high also in defoliated and shaded (TD1 and TD2) treatments. In 2011, it was observed that all the norisoprenoids considered were higher in TD and some others, such as ethoxy actinidol 1 also increased in the TD1 and TD2 treatments. The ND1L treatment showed lower contents for ethoxy actinidol 1, vitispirane and TDN. In 2012, a greater quantity was observed in TD for the unknown norisoprenoid, ethoxy actinidol 1 and vitispirane; ethoxy actinidol 1 was also more abundant in the TD1 treatment. In 2013, only vitispirane was higher in TD, and ethoxy actinidol 1 for TD1 treatment, while ethoxy actinidol 1 was present in less quantities in the ND1L treatment. Chardonnay wine seems to be highly influenced by the agronomic interventions on the canopy, indeed, it is clear that for the main norisoprenoids the total defoliated treatment (TD) was often higher followed by the artificial shading and defoliated treatments. The boxplots of norisoprenoids for each year and treatment are included in the supplementary material (**Supplementary Figure 3**).

##### 3.3.3.2 Pinot noir

Conversely, Pinot Noir wine seems to be less influenced by the treatments (**Figure 7**), although showing similar trends to that seen for Chardonnay, in all the years surveyed ethoxy actinidol 1 was prevailing in the defoliated treatment. In 2010 all compounds except TPB-1 were prevailing in the TD2 treatment while in 2011 only TDN was highest in this treatment. In 2012 vitispirane was the highest in TD treatment while ethoxy actinidol 1 and the unknown norisoprenoid were the lowest in ND1L treatment. In the 2010 and 2013 vintages, safranal, ethoxy actinidol 1 and the unknown norisoprenoids were the highest in the TD1 treatment. In 2013 also the unknown norisoprenoid were highest in the TD treatment as well. The boxplots of norisoprenoids for each year and treatment are reported in the supplementary material (**Supplementary Figure 4**).

## 4 Discussion

### 4.1 Effect of meteorological conditions

The results obtained in this study highlight the influence of temperature and water availability on grapevine yield, musts quality and on the composition of aromas in wine. This confirms the results obtained by other authors that underlined the effect of meteorological conditions on grape and wine quality (Mariani et al., 2009). The average weight of the bunches was higher when water needs were satisfied; 2010 and 2011 were, indeed, characterised by lower water stress indices (WSTR and ΔET) and the highest values of ABW. The grapevine yield positive response to higher water availability was confirmed by other authors (Ramos and Mulligan, 2005; Ramos and Martínez-Casasnovas, 2006; Intrigliolo et al., 2016; Pérez-Álvarez et al., 2021; Ohana-Levi et al., 2022). On the other hand, water scarcity in 2012 lead to a reduction of the bunch weight, causing in turn the typical phenomena of sugar and acidity concentration (Intrigliolo et al., 2016; Pérez-Álvarez et al., 2021) and pH level increase (Reynolds and Naylor, 1994).

Previous studies reported how the ripening of grapes for sparkling wine production was positively influenced by a cool climate (Jones et al., 2014). The optimum temperature for the accumulation of malic acid during grape ripening was estimated to be around 20°C (Lakso and Kliewer, 1975), while the negative correlation between high temperatures and MA content after véraison was reported by many studies (Rienth et al., 2016; Blank et al., 2019). Preserving malic acid degradation made it possible to contrast the decrease of the acidic level in must (Volschenk et al., 2006), which is a desirable condition for sparkling wine production (Jones et al., 2014). In the present study, the positive effect of cooler temperatures on must quality was revealed in 2011, when high values of indicators of suboptimal temperature and optimal temperature were recorded. This led in 2011 to an early ripening (1 August – 12 August), while preserving the must sugar-acid balance, and a low pH. On the contrary in 2010, where similar values for meteorological indicators were reported, the reference sugar level was reached later (24 August - 6 September), leading to lower acidic levels, and higher pH levels. Moreover, the year 2010 reported the highest total amount of cumulated precipitation between berry set and harvest. The low level of titratable acidity and the high value of pH in musts can therefore be related to the effect of dilution of acidic components (Keller, 2006) and the increase of K absorption (Marais et al., 1992) caused by water absorption. The negative effect of high temperatures on the must acidic content was evident in 2013, when a value of the stress caused by heat waves (33STR) was recorded. This was particularly evident in the case of malic acid in Chardonnay musts that showed low values during this vintage. Many previous studies reported the increase of malic acid degradation kinetics when high temperatures were recorded (Michelini et al., 2021).

### 4.2 The effect of leaf removal and artificial shading

Leaf removal and artificial shading effects were differentiated by year and cultivar. In general, years characterized by satisfying water availability and cooler temperatures (i.e., 2010 and 2011) emphasized the difference in ripening delay between the non-artificial shaded and the artificial shaded treatments. This is in agreement with previous papers that reported how shading makes it possible to slow down grape maturation (Caravia et al., 2016; Ghiglieno et al., 2020). The effect of no defoliation and artificial shading on acidity preservation was more evident in years characterized by high temperatures and high-water stress (i.e., 2012 and 2013). This can be related to the positive relationships between malic acid degradation and high temperature and water scarcity (Blank et al., 2019) that suggest the need for shading during warm and dry vintages. In the case of ABW and pH there was no unique response.

The analysis of the main norisoprenoids showed how the canopy treatments greatly affect their quantity (boxplots in **Supplementary Figures 3, 4**). A general increasing effect of total defoliation can be highlighted, especially concerning Chardonnay. Even though Oliveira et al. (2004) reported that carotenoid contents were consistently higher in grapes in the shade than in those exposed to direct sunlight, the positive effect of exposure on norisoprenoids was underlined by many previous studies (Marais et al., 1991; Marais et al., 1992; Asproudi et al., 2016; Asproudi et al., 2020). The results obtained in the present study, in terms of TDN content in wines, showed that this aromatic compound, even if not always in a statistically significant way, was produced in larger quantities in TD while the shaded theses TD1 and TD2 contained less TDN; this behavior was clearly visible in Chardonnay and in two vintages of Pinot noir, and would suggest that it is the light that most influences the biosynthesis of this compound. TDN with a very low olfactory threshold (2 µg l^-1^), can sometimes represent a problem, especially in sparkling wines intended for a long period of aging, because it increases over time. It has been seen that the olfactory threshold of TDN in sparkling wine is slightly higher and goes from 2.1 µg l^-1^ in still Riesling to 4.0 µg l^-1^ in sparkling Riesling (Ziegler et al., 2019); however, this is a compound to pay attention to when deciding on agronomic practices. Several glycosylated precursors have also been reported to originate these compounds during fermentation and wine ageing, through acid-catalysed reactions, and these reactions are certainly promoted in a wine with a low pH, such as sparkling wine (Schneider et al., 2001; Janusz et al., 2003).

The content in vitispirane was always much higher in TD theses and also in this case, although in Pinot noir there was no statistical proof, the defoliated and shaded theses presented a lower content of this compound. The olfactory descriptor of vitispirane is “camphor” or “eucalyptus”. Some studies (Silva Ferreira and Guedes de Pinho, 2004) showed that in aged wines the presence of vitispirane can reach the threshold of perception (800 µg l^-1^) and, consequently, that it participates in the aroma of wine. As with the TDN, pH and temperature are the two factors that have the greatest influence on its formation over time.

(E)-1-(2,3,6-trimethylphenyl) buta-1,3-diene (TPB-1) was tendentially higher in the TD and less present in the shady theses TD1-TD2; this compound, like TDN at high concentrations, could lead to an unpleasant chemical note at higher content (40 ng l^-1^ sensory threshold) (Janusz et al., 2003). The behavior of actinidols and their ethoxy forms was very similar to that seen previously with a higher content in TD, a little lower in the shaded theses and even lower in the not defoliated ones. For this compound the situation was very clear in both Chardonnay and Pinot Noir. Actinidols have an odor that has been described as camphoraceous or as woody and resinous, but their contribution to wine aroma is considered limited at best, as their concentrations are usually much lower than their detection threshold. Perhaps the corresponding ethyl ethers that have been found with the GC-O technique by Schneider et al. (2001), with fruity, citric and eucalyptus notes offer a greater contribution to the aroma.

Another compound that has been increasingly found in TD treatments and less in the other not defoliated and shaded ones was safranal. This compound is responsible for the characteristic smell of saffron and can in some cases exceed the olfactory threshold and be considered too intense, leading to possible depreciation in sparkling wine. It is known that the principal monoterpene glycoside precursor of safranal in saffron is picrocrocin (Zougagh et al., 2006). We don’t know if picrocrocin is a precursor also in wine or if safranal is formed starting from some other precursor or by the rearrangement of some other molecules, but previous studies reported that the content in this compound tends to increase over time, especially at low pH and if the temperature during the storage of the wine is high. Very similar trends in both Chardonnay and Pinot noir, with high levels in the TD thesis and less content in other thesis, were also observed for an unidentified norisoprenoid. On the other hand, we did not observe any clear treatment effects for β- damascenone.

## 5 Conclusions

The results obtained in the present study showed the importance of canopy management, in terms of defoliation and shading, on must and wine quality. This increases our knowledge about the effect of different defoliation and artificial shading application in relation to meteorological condition, supporting the management decision-making in the Franciacorta wine-growing area. The results obtained with no defoliation and artificial shading in the preservation of the acidic composition in warmer vintages suggest that defoliation activities should be calibrated in relation to the meteorological trends, without standardized procedures. This is particularly relevant in the case of sparkling wine production, where the acidic composition is essential to determine wine quality. The enhanced values of some norisoprenoids obtained with the total defoliation treatment represent a further element to direct defoliation and shading strategies. The very low sensory thresholds of some norisoprenoids place them among the important compounds for the final characteristics of the wine. Moreover, the concentrations of these aromatic compounds tend to increase during ageing, especially in sparkling wines, and can reach levels above the threshold, thus developing the characteristic aroma of aged wine, which is sometimes a detrimental wine quality. For example, TDN and TPB-1 found in wine, have a pleasant aroma at low concentrations, but they can reduce the quality of the wine at high concentrations, and it is assumed that other compounds such as safranal, if present at high concentrations, may also be undesirable. However, further studies are needed to establish the olfactory threshold of this compounds in the wine, and which are their precursors in the sparkling wine base before refermentation.

## Data availability statement

The raw data supporting the conclusions of this article will be made available by the authors, without undue reservation.

## Author contributions

IG, SC, GC, LV and FM contributed to the conception and design of the study. IG, SC, GC and UV performed the experiments and analyzed the data. IG, SC, GC and MG-A organized the databases. IG, SC and MG-A performed the statistical analysis. IG, SC and GC wrote the first draft of the manuscript. IG, SC and MG-A prepared figures and tables UV, LV and FM reviewed the drafts of the manuscript. All authors contributed to the article and approved the submitted version.
