# Supplementary material for: Impact of meteorological conditions, canopy shading and leaf removal on yield, must quality, and norisoprenoid compounds content in Franciacorta sparkling wine
Source: Front Plant Sci. 2023 May 17;14:1125560. doi: 10.3389/fpls.2023.1125560 (PMC10229778; doi:10.3389/fpls.2023.1125560)
Supplement: Supplementary file 1 [file DataSheet_1.docx]

Supplementary Material

# Supplementary Figures


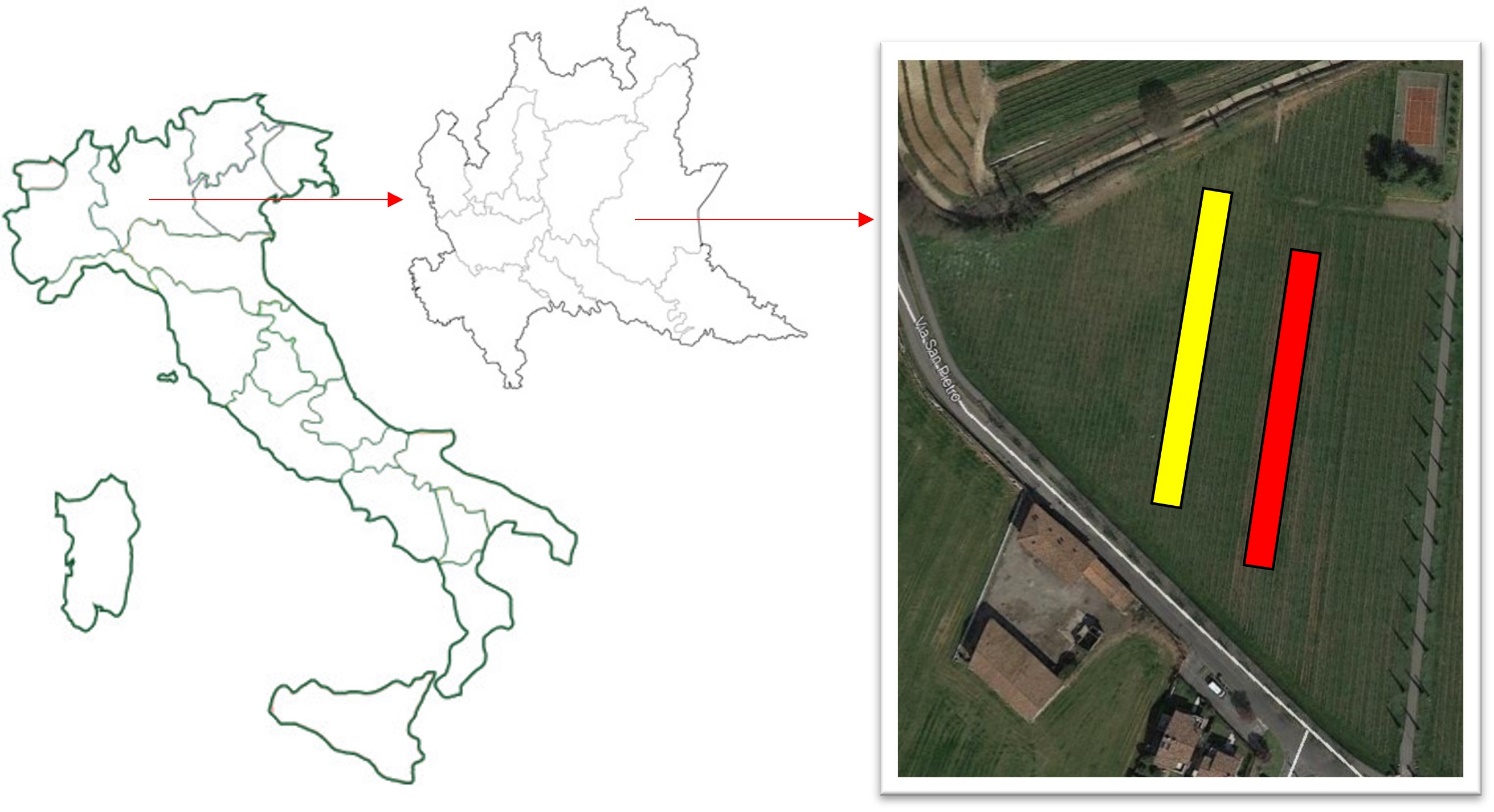


Supplementary Figure 1: Experimental vineyard location and cultivars position within the vineyard; the yellow area indicates Chardonnay, the red area indicates Pinot noir.


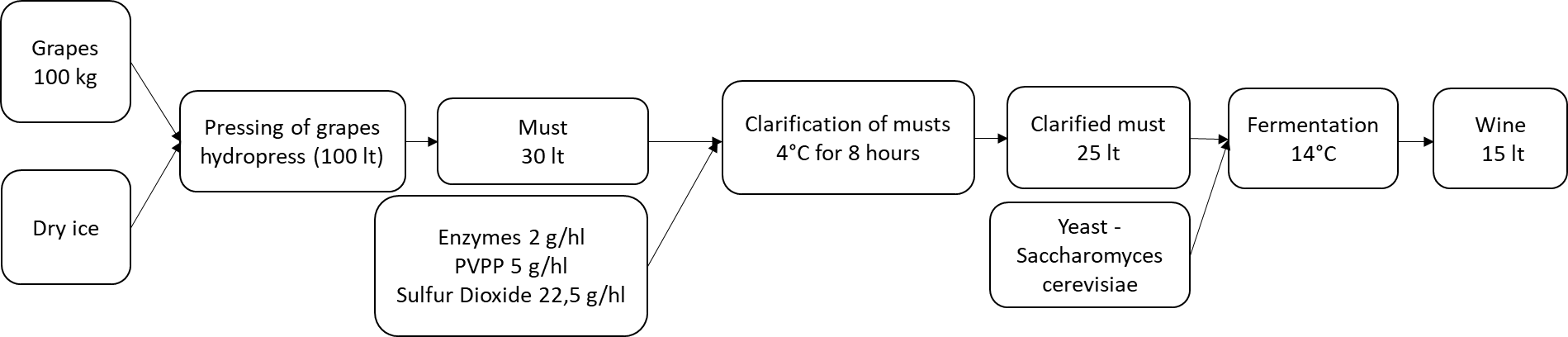


Supplementary Figure 2: Diagram describing the standardized winemaking stages carried out for microvinification


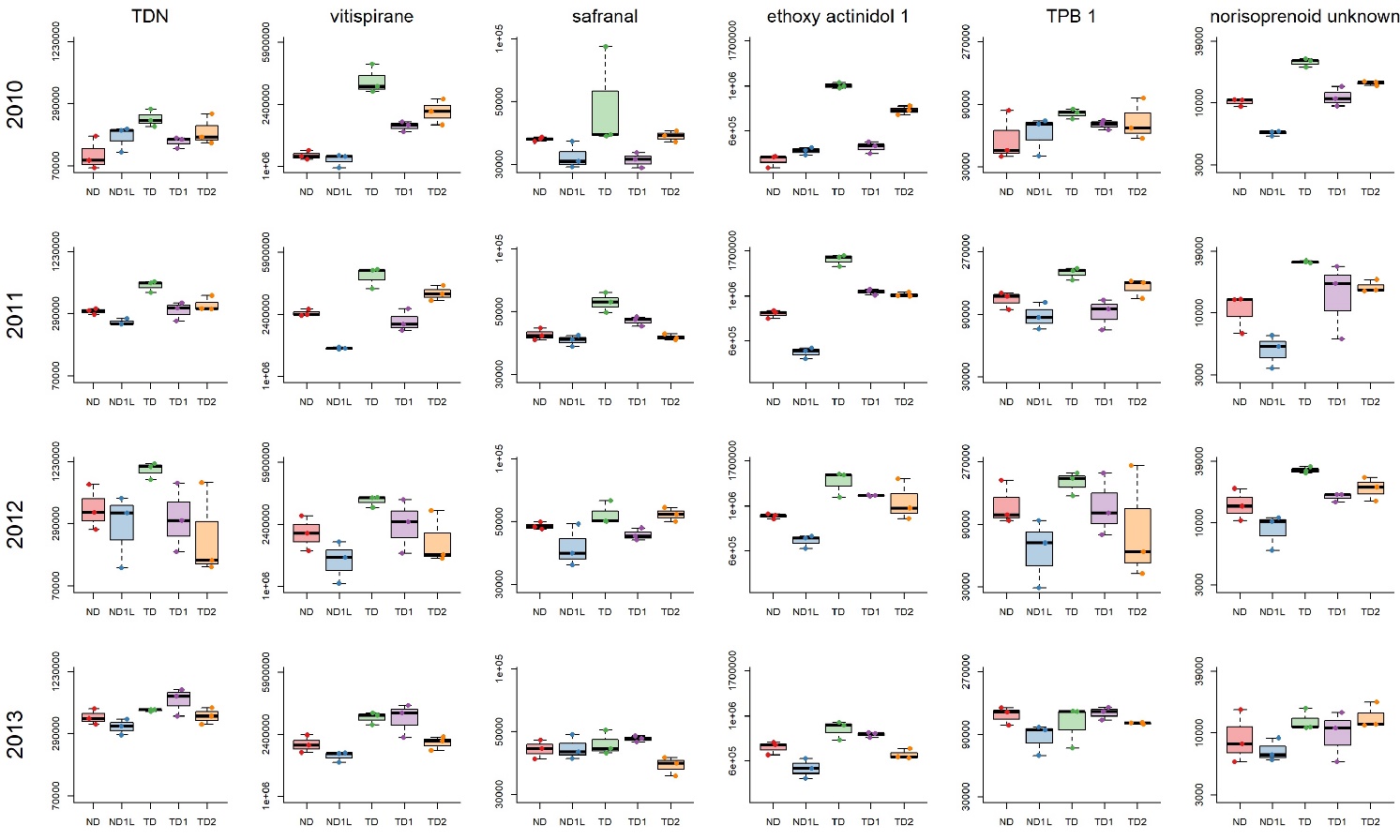


**Supplementary Figure 3**: Boxplot showing the content of norisoprenoid for each treatment in the 4 different vintages for Chardonnay. Values expressed as peak areas and the data is on logarithmic, although the labels shown on the y-axis correspond to their raw values for readability. Each column is referred to one variable (each variable is reported in the upper part of the graph); each row is referred to one year (each year is reported in the left part of the graph).


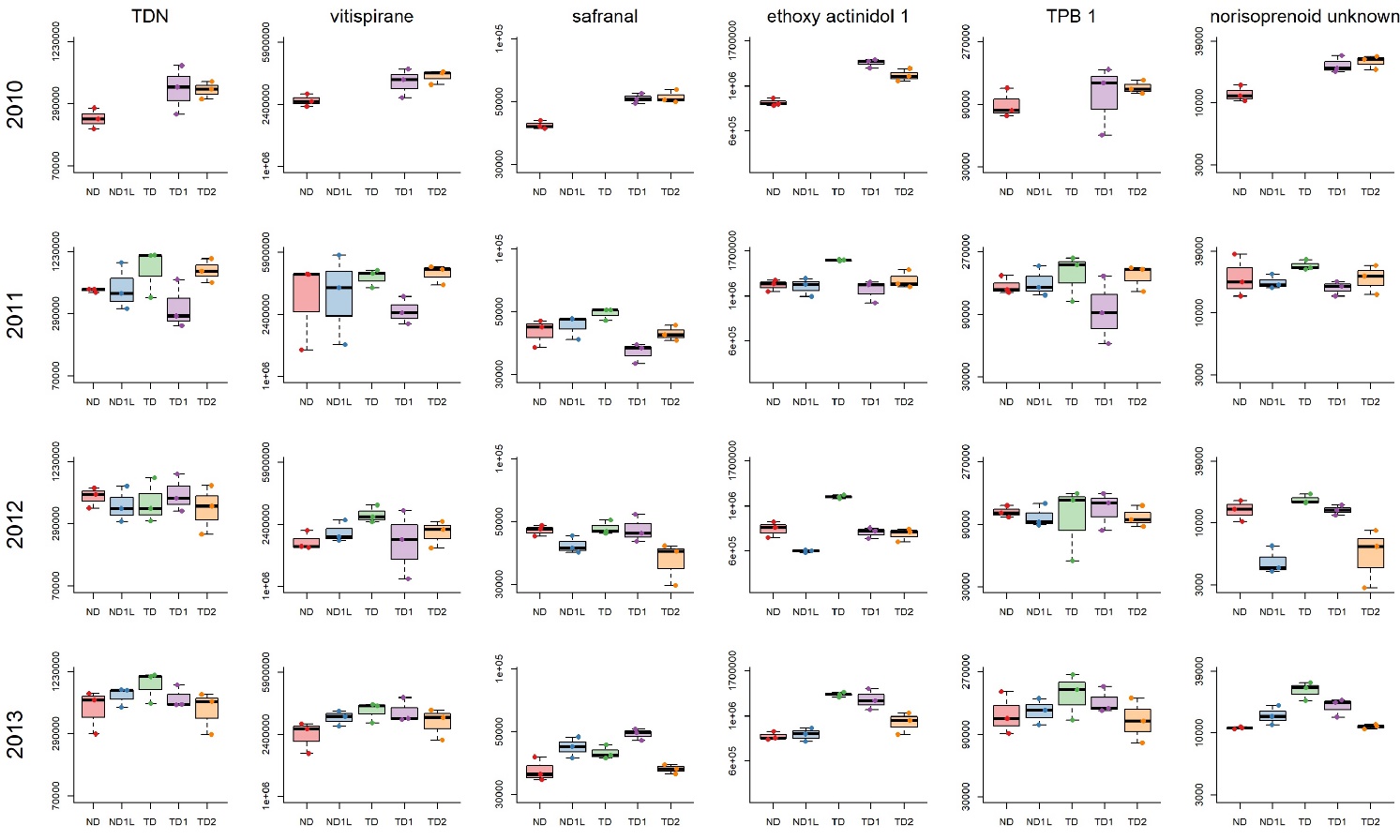


**Supplementary Figure 4**: Boxplot showing the content of norisoprenoid for each treatment in the 4 different vintages for Pinot noir. Values expressed as peak areas and the data is on logarithmic, although the labels shown on the y-axis correspond to their raw values for readability. Each column is referred to one variable (each variable is reported in the upper part of the graph); each row is referred to one year (each year is reported in the left part of the graph).
